# Epidemiology, clinical presentation and outcome of human mpox in Rivers State, Nigeria during the 2022-23 outbreak: an observational retrospective study

**DOI:** 10.11604/pamj.supp.2025.50.1.46067

**Published:** 2025-03-03

**Authors:** Chizaram Onyeaghala, Abiye Somiari, Ujile Ichechiek, John Ogan, Hope Avundaa, Odianosen Ehiakhamen, Bolaji Ibiesa Otike-Odibi, Omosivie Maduka, Datonye Alasia

**Affiliations:** 1 Department of Internal Medicine, University of Port Harcourt Teaching Hospital, Port Harcourt, Nigeria,; 2 Department of Community of Medicine, University of Port Harcourt Teaching Hospital, Port Harcourt, Nigeria,; 3 Nigeria Centre for Disease Control and Prevention, Abuja, Nigeria

**Keywords:** Mpox, epidemiology, clinical presentation, Rivers State, Nigeria

## Abstract

**Introduction:**

limited studies describe the epidemiology, clinical presentation, and outcome of mpox in Rivers State, Nigeria.

**Methods:**

an observational retrospective study of suspected mpox cases seen between October 21, 2021, and April 30, 2023, at the University of Port Harcourt Teaching Hospital, Rivers State, Nigeria. Laboratory confirmation was by qPCR testing of skin swabs, or crust samples. A data extraction form was used to obtain sociodemographic and clinical data. The primary outcome was the rate of hospitalization and death. Data was analyzed using IBM SPSS version 25. A p-value of <0.05 was considered statistically significant.

**Results:**

of 35 participants, 24 (68.6%) were males with a mean age [standard deviation] of 33.7 (± 9.9 years), and 5 (14.3%) had HIV infection. The majority (n=33, 94.3%) reported being in a heterosexual relationship with a household identified as the probable place of contact (n=13, 72.2%). None of the patients reported contact with animals or a travel history. The most common symptoms were skin rash (35, 100%), followed by fever (26, 74.3%), and fatigue (26, 74.3%). Concomitant varicella zoster virus infection (n=6, 17.1%), hospital admission (n=28, 80%), and death (n=3, 8.6%) were observed. There was a statistically significant association between mortality and patients with HIV (p = 0.002).

**Conclusion:**

the 2022-23 mpox outbreak in Rivers State predominantly affected young adults and was characterized by human-to-human transmission and typical febrile rash syndrome with an association between mpox mortality and HIV infection. Targeted public health interventions are needed to control the spread of mpox in Nigeria.

## Introduction

Mpox (formerly monkeypox) is an emerging zoonotic infection caused by the monkeypox virus (MPXV), a DNA (deoxyribonucleic acid) virus of the orthopoxvirus genus and the family poxviridae [1-3]. First described in captive research monkeys in Copenhagen, Denmark in 1958 and later found in humans in 1970 in the Democratic Republic of Congo (DRC) [3,4]. There are 2 distinct clades of the virus: clade I (subdivided into clades Ia and Ib) which is responsible for the 2024 mpox upsurge in DRC and some parts of East Africa [5,6], and Clade II (subdivided into IIa and IIb) with the IIb strain responsible for the 2022 global outbreak. The Clade II (previously known as the West African group) typically causes milder clinical disease and fewer fatalities than Clade I (previously known as the Central African (Congo Basin) group) which is associated with a more severe clinical course and higher mortality [7,8]. As of June 30, 2024, 99,176 confirmed cases and 208 deaths were reported in 116 countries across the globe [9].

The mpox disease landscape has been characterized by evolving epidemiology and clinical presentation, with repeated outbreaks. During the 2017 reemergence in Nigeria, there was a notable demographic change to more urban, young adults (30 to 40 years), compared to previous cases of children (less than 17 years) in rural communities [2,10]. Likewise, the 2022 global mpox outbreak revealed an unprecedented change from a predominantly zoonotic transmission characterized by typical febrile vesiculopustular eruptions marked in the face and limbs [10] to sexual transmission among men who have sex with men (MSM) characterized by atypical anogenital lesions [11,12]. In 2024, DRC and some East African countries witnessed an upsurge in mpox cases leading to the declarations of mpox as a public health emergency of continental security (PHECS) for the first time on August 13, 2024 by the Africa Centres of Disease Control and Prevention (ACDC) and a public health emergency of international concern (PHEIC) on August 14, 2024 by the World Health Organization (WHO) for the second time in two years [13].

The exact animal(s) that serve as reservoir hosts for MPXV is not known, but rodents and other nonhuman primates have been implicated [7]. Transmission of MPXV occurs through direct contact with infected animals, and from person to person through direct contact or indirect contact with contaminated surfaces [7,14-17]. The disease incubation period is usually 5-21 days (average of 12 days) but may range from 1 to 30 days depending on the infecting clade of the virus, host immune status, and transmission route [14-17]. Most patients with mpox recover within 3 to 4 weeks of onset of skin rash either spontaneously or requiring supportive treatment. However, severe illness with complications such as pneumonia, encephalitis, sepsis, and secondary bacterial infections can sometimes result in death among the elderly, children, pregnant women, persons with chickenpox coinfection [18], and those with advanced HIV disease [19] if not appropriately managed.

There have been limited studies aimed at filling the knowledge gaps in the epidemiology, clinical presentations, and outcome of human mpox during the 2022 global outbreak in Nigeria [20,21]. Rivers State reported the 2nd highest number of documented mpox cases during the 2022 outbreak in Nigeria. A genomic surveillance investigation identified Rivers State as the “epicentre” of mpox resurgence in Nigeria with the occurrence of cryptic human-to-human transmission of MPXV approximately three years before the detection of the first case in September 2017 [22]. This study aimed to describe the epidemiology, clinical presentations, and outcome of human mpox cases in Rivers State, Nigeria, during the 2022-23 outbreak.

## Methods

**Study design, study site, and study participants:** we conducted a retrospective observational study of all persons with suspected mpox managed at the mpox treatment center and outpatient clinic in the University of Port Harcourt Teaching Hospital, Port Harcourt, Rivers State, Nigeria, between October 21, 2021 and April 30, 2023. This tertiary hospital has 782 beds with a 20-bed isolation and treatment centre where mpox and other high-consequence infectious diseases are managed. This referral centre serves the entire Rivers State and the neighbouring states. Rivers State has 23 Local Government Areas with an estimated population of 8.6 million [23] and is bordered by the Atlantic Ocean, Bayelsa, Akwa-Ibom, Imo, and Abia States.

All patients who met the criteria for suspected mpox cases per the Nigeria Centre for Disease Control and Prevention (NCDC) guidelines were eligible for enrolment into this study. The NCDC guidelines for mpox case definition applied were [24]: (1) a suspected case of mpox was defined as “any person presenting with a history of sudden onset of fever, followed in a few days by a vesiculopustular rash occurring mostly on the face, palms, and soles of the feet;” (2) a confirmed case of mpox was defined as “any suspected case with laboratory confirmation of mpox by virus isolation or PCR;” and (3) a probable case of mpox was defined as “any suspected case with epidemiological linkage to a confirmed case in which laboratory testing could not be carried out”. Laboratory confirmation of mpox and varicella-zoster virus infection with real-time polymerase chain reaction (RT-PCR) assay of swabs from the exudate of vesicular or pustular lesions and/or crust skin specimen was done centrally at the National Reference Laboratory of the NCDC, Gaduwa-Abuja, Nigeria.

The detection of mpox and varicella zoster virus is based on TaqMan assay with primers and probes targeting mpox and varicella zoster virus with the same thermal profile in one run. The two targets are detected in the FAM channel, using TaqMan buffer and US Centers for Disease Control and Prevention in-house primers and probe master mix separately prepared for mpox, varicella-zoster virus, and RNase P. mpox-positive samples that met eligibility criteria for sequencing (cycle threshold value < 30 and sample availability) were screened for DNA concentration and quality (total DNA >500ng and absorbance ratio >1.8 260/230 and 260/280). Those with good quality were transferred to the African Centre for Excellence for Genomics of Infectious Diseases (ACEGID), based at Redeemer´s University, Ede, Nigeria where sequencing, genome assembly, genomic dataset curation, phylogenetic analysis, and modeling APOBEC3-mediated evolution were performed [22].

**Data collection:** data was extracted using an MS-Excel-based data extraction sheet adapted from the WHO mpox case reporting form version 3 dated 22 December 2022. Data extracted included sociodemographic information (age, gender, occupation, sexual orientation, and potential MPXV transmission routes such as animal contact, close contact, and sexual exposure), clinical manifestations, presence of comorbidities (e.g., concomitant chickenpox coinfection), HIV status, laboratory information, and treatment received. Additionally, vital signs documented at presentation (axillary temperature, respiratory rate, oxygen saturation), complications, and outcomes were noted. Patients were managed per NCDC guidelines [24]. Patients with HIV infection were categorized as early HIV disease (WHO stage 1 or 2 disease with CD count > 200 cells per mm3) or advanced HIV disease (WHO stage 3 or 4 disease, or CD4 count <200 cells per mm3) [25].

Complications associated with mpox included skin complications (e.g. secondary bacterial skin infection, scarring, and skin/genital oedema), mucosal complications (e.g. pharyngotonsillitis, anogenital ulcers, ocular injuries, and urethritis), and systemic complications (e.g. sepsis, encephalitis, and pneumonia). The severity of mpox was defined from a similar study conducted in Nigeria [21]. Severe disease was defined as any death associated with mpox or any person with a life-threatening complication (i.e. any of the following: sepsis, encephalitis, severe pneumonia with hypoxaemia or oxygen dependence, and retropharyngeal abscess with airway obstruction). Moderate disease was a person with any non-life-threatening skin, mucosal, or systemic complications. Mild disease was a person with no reported complication. Management outcomes were categorized as hospital admission (outpatient vs hospitalized) or survival (survived vs died). Duration of hospitalization was the number of days until discharge or death, whichever came first.

**Statistical analysis:** the data extracted was exported into IBM Statistical Product and Service Solutions (SPSS) version 25 for analysis. Continuous variables were summarized as mean ± standard deviation (SD) when normally distributed or as median and interquartile ranges (IQR) when skewed. Categorical variables were summarized as frequencies and proportions. Missing values were reported as unknown. Associations between categorical variables were done using Fisher´s exact test. We considered a p-value of < 0.05 (two-sided) as statistically significant.

**Ethical considerations:** ethical approval was obtained from the Research and Ethics Committee, University of Port Harcourt Teaching Hospital (NHREC/UPTHHREC/02/2023). Patient consent was not required as it was a retrospective study. The study was conducted under the declarations of Helsinki and followed local guidelines for medical research involving human participants.

## Results

A total of 35 suspected mpox cases were managed in the mpox treatment centre or outpatient clinic during the 2022-23 outbreak. Of the 35 patients, 24 (68.6%) were males and the mean age [± standard deviation] was 33.7 (± 9.9 years). Up to a third of the patients, 12 (34.3%) were traders. All participants resided in urban or semi-urban settings. Among the 35 cases documented, 5 (14.3%) were HIV seropositive, 14 (40.0%) were seronegative and 16 (45.7%) of them had unrecorded HIV results. Of the 5 documented HIV seropositive patients, 3 (60%) of them had advanced HIV disease (AHD), and 4 (80%) were newly diagnosed with HIV and had started ART within 2 weeks. All CD4 counts were measured qualitatively (i.e., ≥ 200 CD4 cells per mm3 vs > 200 cells per mm3) using the VISITECT lateral flow assay point-of-care testing. None of the participants had available HIV viral load results. Of the 3 cases with AHD, screening for opportunistic infections such as tuberculosis and cryptococcal meningitis were offered as part of the AHD package of care using lateral flow urine lipoarabinomannan and serum cryptococcal antigen testing respectively, and were negative.

**Figure 1:**
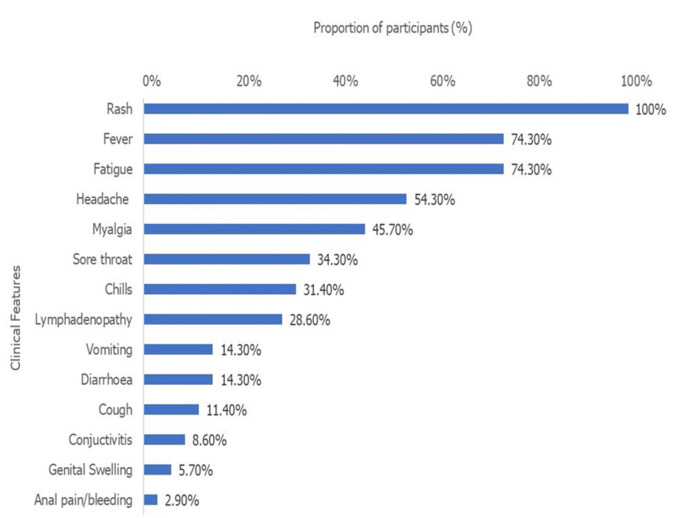
clinical features of mpox

Regarding sexual relationships, 33 patients (94.3%) self-reported being in a heterosexual relationship and one patient (a 12-year-old male) was not sexually active. The sexual orientation of one of the cases was not documented and none identified as homosexual. In about half of the cases, 18 (51.4%) had reported a history of contact with a confirmed case, while 13 (72.2%) of these reported contacts occurred within households. None of the patients reported a history of travel out of the state or country, and none reported contact with animals (Table 1). The most common symptoms reported by the patients were skin rash (35, 100%), followed by fever (26, 74.3%), fatigue (26, 74.3%), and headache (19, 54.3%) (Figure 1). The first rash appeared on the face in 33 cases (94.3%) and the anogenital region in 2 of the patients (5.7%). The type of skin rashes observed were pustules (32, 91.4%), papules, (27, 77.1%), and vesicles (21, 77.1%). The body parts where the skin lesions were commonly located included in order of frequency: the trunk (35, 100%), limbs (35, 100%), genitals (29, 82.9%), face (27, 77.1%), and scalp (8, 22.9%). About half of the participants, 18 (51.4%) reported having symptoms that lasted 7 or more days before presentation.

**Table 1:** sociodemographic and epidemiological characteristics of suspected human mpox cases in Rivers State, Nigeria during the 2022-23 outbreak

Characteristic (n = 35)	Frequency	Percentage (%)
**Age (years)**		
< 20 years	2	5.7
20 – 40 years	22	62.9
> 40 years	11	31.4
**Sex**		
Female	11	31.4
Male	24	68.6
**Occupation**		
Unemployed	1	2.9
Police/security officer	2	5.7
Civil servant	7	20.0
Student/youth corp member	12	34.3
Trader	13	37.1
**HIV status**		
HIV positive	5	14.3
HIV negative	14	40.0
Not recorded	16	45.7
**CD4 count (N=5)**		
Less than 200 cells/mm^3^	3	60.0
≥ 200 cells/mm^3^	2	40.0
**Sexual orientation**		
Heterosexual	33	94.3
Not sexually active	1	2.9
Homosexual	0	0.0
Not recorded	1	2.9
**History of contact with a case**		
Yes	18	51.4
No	2	5.7
Unknown	15	42.9
**Probable place of contact with case (N = 18)**		
Household	13	72.2
Transport	2	11.1
Work place	2	11.1
Social gathering	1	5.6
**History of contact with animals**		
No	35	100.0
History of travel		
No	35	100.0

A total of 28 (80%) of the patients were admitted to the hospital with 18 (64.3%) staying in the hospital between 2 and 21 days, with a median of 5 (IQR of 5-8) days. At presentation, 9 (25.7%) of the cases had temperatures of 38° celsius or more, 10 (28.6%) had respiratory rates of more than 20 cycles per minute, and 11 (31.4%) of them had oxygen saturation (SPO2) levels of less than 95% (Table 2). Ten patients (28.6%) received the antiviral (Acyclovir) and all patients received broad-spectrum antibiotics (35, 100%). Amoxicillin-clavulanic acid was the most commonly prescribed antibiotic to cover for secondary bacterial infections. No patient received specific mpox therapeutics (tecovirimat, cidofovir, brincidofovir, vaccinia immune globulin intravenous) and none received an mpox vaccine.

**Table 2:** clinical characteristics of suspected human mpox cases seen in a treatment centre in Rivers State, Nigeria during the 2022-23 outbreak

Characteristic (N = 35)	Frequency	Percentage (%)
**Hospital admission**		
Hospitalized	28	80.0
Outpatient	7	20.0
**Duration of hospitalization (N = 28)**		
< 7 days	18	64.3
≥ 7 days	10	35.7
**Temperature at presentation**		
< 38 Celsius	26	74.3
≥ 38 Celsius	9	25.7
**Respiratory rate at presentation**		
≤ 20 cycles per minute	25	71.4
> 20 cycles per minute	10	28.6
**SPO2 at presentation**		
≥ 95%	24	68.6
< 95%	11	31.4
**Use of antiviral agents**		
Yes	10	28.6
No	25	71.4
**Specific antiviral agents used (N = 10)**		
Acyclovir	8	80.0
Valacyclovir	2	20.0
**Antibiotics received**		
Amoxicillin/clavulanic acid	33	94.3
Ceftriaxone	1	2.9
Levofloxacin	1	2.9
**Complications**		
Yes	8	22.9
No	27	77.1
**Type of complications recorded**		
Skin infection	8	22.9
Scrotal oedema	2	5.7
Pharyngotonsillitis	3	8.6
Ocular injuries	1	2.9
Urinary retention	1	2.9
Necrotizing genital ulcers	3	8.6
Sepsis	3	8.6
Pneumonia	2	5.7

**Figure 2:**
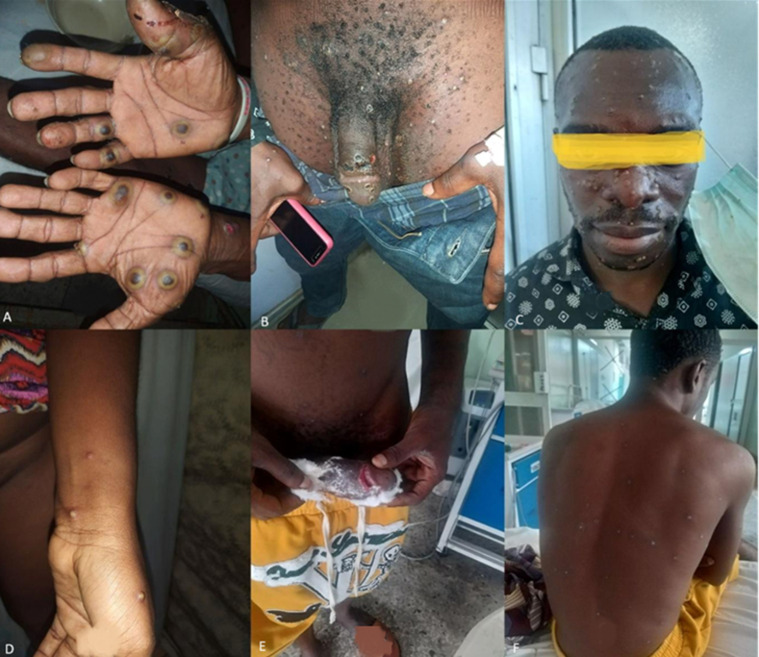
(A-F) clinical images of skin and senital mpox lesions

A total of eight or 22.9% of patients were noted to have one or more complications. The skin complications reported were secondary bacterial skin infections (8, 22.9%), and scrotal oedema (2, 5.7%). The mucosal complications were pharyngotonsillitis (3, 8.6%), ocular injuries (1, 2.9%), and urinary retention (1, 2.9%). Among all patients with complications, necrotizing and genital ulcers were observed in 3 or 8.6% of all patients (Figure 2), all of whom had advanced HIV infection. The systemic complications reported were sepsis (3, 8.6%) and pneumonia (2, 5.7%) (Table 2). Of the 35 suspected cases, 24 (68.6%) of the cases were laboratory-confirmed mpox positive cases. Of these laboratory-confirmed cases, 6 (25%) of them also tested positive for Varicella zoster virus (VZV). Amongst the traditional sexually transmitted infections, only syphilis screening was offered to patients (6, 17%) using VDRL and all returned negative. Of the 35 patients, 3 (9%) of them died (2 males and one female), all aged between 40 and 50 years. All 3 cases were hospitalized, were HIV seropositive, and had AHD. One of the fatalities was also positive for concomitant VZV infection. Possible causes for the fatalities recorded were sepsis and severe pneumonia. Among all participants, mpox-VZV coinfection was not associated with death (p= 0.442), however, HIV infection among the cases was associated with a fatal outcome (p= 0.002) (Table 3).

**Table 3:** relationship between Mpox-VZV and Mpox-HIV coinfection and outcome of cases seen in a treatment centre in Rivers State during the 2022-23 outbreak

	Alive	Dead	P-value
	N (%)	N (%)	
**Mpox-VZV coinfection**			
Coinfected	5 (83.3)	1 (16.7)	0.442^b^
Not coinfected	27 (93.1)	2 (6.9)	
**HIV status**			
Positive	2 (40)	3 (60)	0.002*^b^
Negative	14 (100)	0 (0)	
Not recorded	16 (100)	0 (0)	

b Fisher’s Exact test; *Significant at 5%

## Discussion

To the best of our knowledge, this is the first paper from Rivers State Nigeria that described in addition to the epidemiology, the clinical manifestations and outcome of mpox. Our study data showed that the 2022-23 mpox outbreak began in the last quarter of 2021 and continued till April 2023 after the WHO had ended the public emergency of mpox. Our findings revealed that mpox predominantly affected young adults driven by human-to-human transmission through sexual and nonsexual (household) contacts. We identified skin rash, fever, fatigue and headache as the commonest clinical presentation of mpox and an association between mortality and HIV infection was noted. The demographic characteristics of the participants in this study are in keeping with studies during the 2017 and 2022 mpox outbreaks in Rivers State and in Nigeria, which predominantly affected young adult men who are urban or semi-urban dwellers [21,26,27].

The mean age of 33.7 years in our study agrees with studies that reported the age group 30-34 years as having the highest number of cases reflecting waning immunity against mpox infection following smallpox eradication in 1980 [21,26,27]. Similarly, the majority of the mpox cases reported during the 2022 outbreak in countries outside of endemic settings were among young urban adults who identify as being male; being gay, bisexual, and other men who have sex with men (GBMSM) [12,28]. We did not report any GBMSM in this study representing a distinct epidemiologic difference between mpox in endemic Nigeria and non-endemic countries. A plausible reason for this could be that the 2022-23 mpox outbreak in Nigeria was not particularly driven among this key population. It could also be due to under-recognition by the surveillance officers and clinicians or that they declined to disclose their exposure due to fear of stigma and victimization since there is a prohibition of same-sex relationships in Nigeria. This could potentially be a public health concern capable of undermining the outbreak response.

We found human-to-human spread to be the major mode of MPXV transmission in Rivers State through sexual and nonsexual physical contact. Although we did not establish epidemiologic risk factors that were independently associated with mpox positivity due to the small sample size recorded in this study, we identified mpox among heterosexual partners similar to a study done in Bayelsa State, Nigeria supporting the role of previous sexual activity and mpox infection [29]. While travel plays a crucial role in propagating infectious disease outbreaks as witnessed during the COVID-19 pandemic [30], we did not report any travel history outside the state or country in the study population. A genomic analysis identified Rivers State as the “epicentre” of mpox resurgence in Nigeria with cryptic sustained human-to-human MPXV transmission largely driven by APOBEC3 protein activity occurring for approximately three years before viral export to the neighbouring state of Bayelsa where the first case was reported on September 2017 [22]. This also aligns with an unpublished report of clusters of predominantly genital “pox-like” lesions managed between May and July 2017 in the University of Port Harcourt Teaching Hospital, Rivers State highlighting the role of genomic epidemiology in complementing classical epidemiology during infectious diseases outbreaks.

Genomic sequencing of mpox-positive samples identified clade IIb non-B.1 sequence (sub-lineage A.2.3) as the predominant circulating variant of MPXV in Nigeria during the 2022-23 outbreak [22]. Although Gao et al. [30] similarly identified clade IIb on phylogenetic analysis of 175 MPXV genomes from several regions, the B.1 lineage was found to be the major variant driving the 2022-23 outbreak in non-endemic countries [22,30]. Contrarily, the 2024 upsurge in mpox cases in DRC has been linked to multiple lineages of clade Ia and the recently discovered clade Ib variants [31]. The reports of mpox among PLHIV coupled with the poor outcome of mpox in PLHIV during the 2022-23 outbreak has led to surveillance integration of these co-viral pathogens especially in endemic settings such as Nigeria to ensure timely public health intervention. Most (80%) of them are newly diagnosed HIV and ART-naive reflecting persisting challenges of limited access to diagnosis, treatment, and prevention in low-income countries such as Nigeria. Up to 60% of PLHIV of the study population had advanced HIV infection mirroring the high burden of advanced HIV disease among PLHIV in Nigeria and other parts of Africa [32].

Our study data reported concomitant chickenpox coinfection reflecting the co-circulation of chickenpox among the young adult population in Nigeria [18,20] posing a diagnostic conundrum for mpox due to similar clinical presentations [33-35]. This is plausible since chickenpox is not a notifiable disease and the Varicella zoster virus vaccine is not part of routine immunization in Nigeria. The high coinfection rate in this study (17.1%) is similar to other Nigerian studies [18,33,36] and differs from the lower rates reported in DRC [33,37]. Similar to [18], we did not observe any statistically significant association of fatal outcomes in the mpox and chickenpox co-infection cohort, although mortality was observed in one of our mpox patients with chickenpox and advanced HIV. The higher rates of hospitalization (80%), varicella zoster virus co-infection (17.1%), and mortality (9%) in the study population which differed from studies done in the non-endemic countries during the 2022-23 outbreak may be attributed to the mode of MPXV transmission, viral clade, sample size differences, host immune response to the virus, and access to medical intervention. The identification of predominantly typical febrile rash syndrome with few reports of unusual localized genital lesions in our cohort emphasizes the place of both sexual and nonsexual (household) contact in the transmission of MPXV in Rivers State in keeping with a similar study [20].

Our patients were managed with supportive measures as per NCDC guidelines [24], as there was no access to mpox-specific therapeutics (tecovirimat, brincidofovir, cidofovir, and vaccinia immune globulin intravenous) or mpox vaccines during the 2022-23 outbreak in Nigeria highlighting issues of global health inequity during outbreaks [38]. In settings where these medical countermeasures were available, the use of tecovirimat showed mixed results particularly in those with advanced HIV with reports of rebound viraemia and resistance following a standard 14-day course therapy [39-42]. mpox vaccination (Modified vaccinia Ankara) began for the first time in Nigeria in November 18, 2024, targeting at-risk populations (health workers particularly clinicians managing mpox cases and support staff; laboratory staff handling samples including surveillance and notification officers; HIV patients, and close contacts of confirmed mpox cases within 42 days of case detection) across seven states based on epidemiologic data. All participants (100%) received empirical broad-spectrum antibiotics for secondary bacterial skin infections and sepsis without evidence of blood/wound swab culture or sepsis biomarkers. In this era of increasing antimicrobial resistance and from the lessons learned from the COVID-19 pandemic, there is a need for institutionalization of antimicrobial stewardship practices during outbreaks.

There was a significant relationship between mpox mortality and patients with HIV infection. In particular, we observed that mpox patients presented with necrotizing lesions, protracted illness, and higher rates of complications such as secondary bacterial skin infections and sepsis. Likewise, a global case series reported necrotizing mpox lesions among patients with advanced HIV during the 2022 outbreak [19]. We observed that one of our patients with treatment-naive advanced HIV disease died within 2 weeks of ART initiation in keeping with the PEPFAR-supported African Cohort Study (AFRICOS) that showed that mortality among people living with HIV with AHD occurred within a few weeks after presenting to care [32]. Clinicians need to be cautious in the timing of ART commencement in mpox patients with advanced HIV infection due to the possibility of an immune reconstitution inflammatory syndrome. A similar observation was reported by clinicians outside of endemic settings [19,43], with Mitja et al. proposing that mpox in people with advanced HIV may be an “AIDS-defining” illness [19]. Our study is limited by its small sample size, which made it difficult to conduct a multivariate analysis. Additionally, the fact that it was a hospital-based study may have selected moderate to severe mpox cases potentially excluding the predominantly mild mpox-positive cases in the community thereby under-diagnosing the true picture of the 2022-23 mpox outbreak in Rivers State. All of this could affect the generalisability of the study. Nevertheless, its contribution to the body of knowledge in mpox clinical-epidemiologic studies in resource-limited settings emphasizes the strength of this study.

## Conclusion

Our study reveals that mpox during the 2022-23 outbreak primarily affected young urban adults driven by human-to-human transmission with an association between mpox mortality and those with HIV infection. The identification of sexual and household contacts as MPXV transmission routes underscores the need for targeted and strategic public health interventions to control mpox transmission in Nigeria. Further studies are needed to fill the substantial knowledge gaps in mpox epidemiology and clinical manifestations in Nigeria.
